# Effect of teduglutide, a glucagon-like peptide-2 analog, in eosinophilic enterocolitis: a case report

**DOI:** 10.3389/fped.2024.1457824

**Published:** 2024-10-08

**Authors:** Shoko Ogawa, Ken-ichiro Konishi, Kiyoshi Tanaka, Hajime Takayasu, Yoshimasa Uematsu, Takashi Ito, Hiroyuki Takahashi, Yusuke Kumamoto

**Affiliations:** ^1^ Department of General-Pediatric Hepatobiliary Pancreatic Surgery, Kitasato University School of Medicine, Kanagawa, Japan; ^2^ Division of Pediatric Surgery, Department of Advanced Medicine, Research and Development Center for New Medical Frontiers, Kitasato University School of Medicine, Kanagawa, Japan; ^3^ Department of Pathology, Kitasato University School of Medicine, Kanagawa, Japan; ^4^ Department of Pathology, Kitasato University School of Allied Health Sciences, Kanagawa, Japan

**Keywords:** glucagon-like peptide-2 analog, immune effect, short bowel syndrome, eosinophilic gastrointestinal diseases, teduglutide

## Abstract

We successfully treated a 4-year-old girl with short bowel syndrome and eosinophilic enterocolitis with teduglutide, a glucagon-like peptide-2 analog. Her eosinophilic enterocolitis was cured without relapse, and we were able to increase enteral nutrition. We found that teduglutide had an anti-inflammatory effect in this patient with eosinophilic gastrointestinal disease associated with short bowel syndrome. This report is the first to describe use of teduglutide in the treatment of eosinophilic gastrointestinal disease.

## Introduction

Inflammatory changes may be seen in the setting of short bowel syndrome (SBS). Several reports have suggested a relationship between SBS and eosinophilic gastrointestinal diseases (EGIDs) (1). Although the mechanism has not yet been elucidated, it is thought to involve impaired gastrointestinal function due to the underlying disease, inflammation due to surgical invasion or intestinal congestion, disruption of the gastrointestinal mucosal barrier mechanism due to ischemia, atrophy of the gastrointestinal mucosa due to prolonged fasting, and effects of postoperative antibiotics on the intestinal microbiota (2, 3).

EGIDs are characterized by eosinophilic inflammation with eosinophilia of unknown cause (4). The pathogenesis of EGID is only partially understood. Histopathological findings in patients with EGID include an excessive number of activated eosinophils with signs of degranulation. Several signals are responsible for activation of eosinophils, including non-specific tissue damage, infection, and allergens. Activated eosinophils produce inflammatory mediators that trigger degranulation of mast cells and release of chemokines, cytokines, lipid mediators, and neuromediators (4).

Recent reports suggest that the glucagon-like peptide-2 (GLP-2) analog teduglutide may have a significant impact in treating SBS (5). Teduglutide, which has been approved for the treatment of patients with SBS in 29 countries as of July 2024, was approved in Japan in 2021. GLP-2 is secreted by enteroendocrine L-cells in the terminal ileum and colon and was discovered to be a key factor in intestinal rehabilitation and growth (6). GLP-2 stimulates the growth of the intestinal mucosa by stimulating proliferation of crypt cells and inhibiting apoptosis of enterocytes (7). Teduglutide improves intestinal absorption of fluids and nutrients, thereby reducing the need for parenteral support (5). However, it is unknown whether teduglutide has a favorable impact on intestinal inflammation in patients with EGID. To our knowledge, this is the first case report to describe the use of teduglutide in the treatment of EGID-associated SBS.

## Case

**Figure 1:**
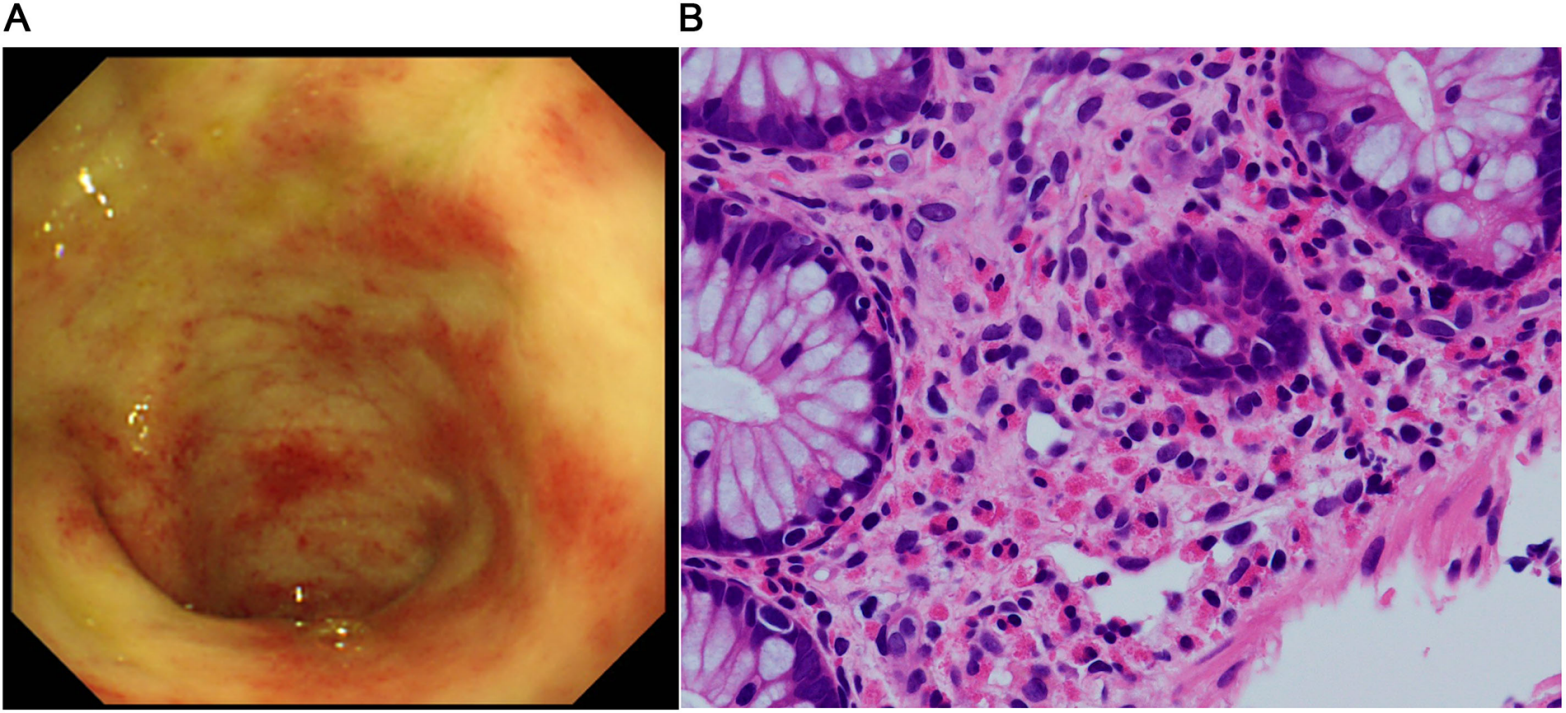
Findings before starting the GLP-2 analog teduglutide. **(A)** Colonoscopic image shows multiple erythematous lesions and pale edematous mucosa with decreased vascularity. **(B)** Pathological image of the mucosal specimen shows superficial abrasion and a high-grade eosinophilic infiltrate in the epithelium (>50 counts/high power field).

**Figure 2:**
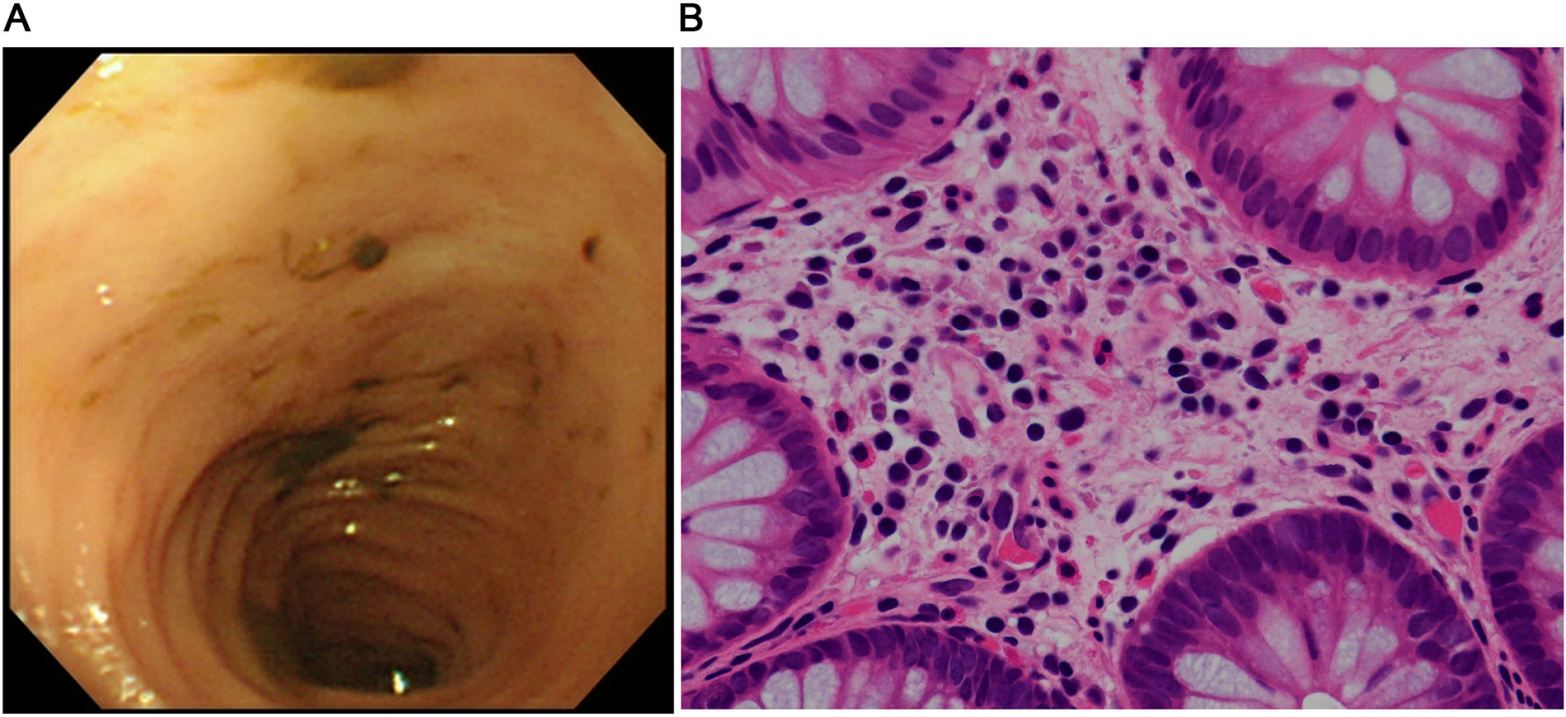
Findings after starting the GLP-2 analog teduglutide. **(A)** Colonoscopic image shows complete resolution of the erythema in the colon. **(B)** Pathological image of the mucosal specimen from the colon shows fewer eosinophils in the epithelium (<10 counts/high power field).

**Figure 3:**
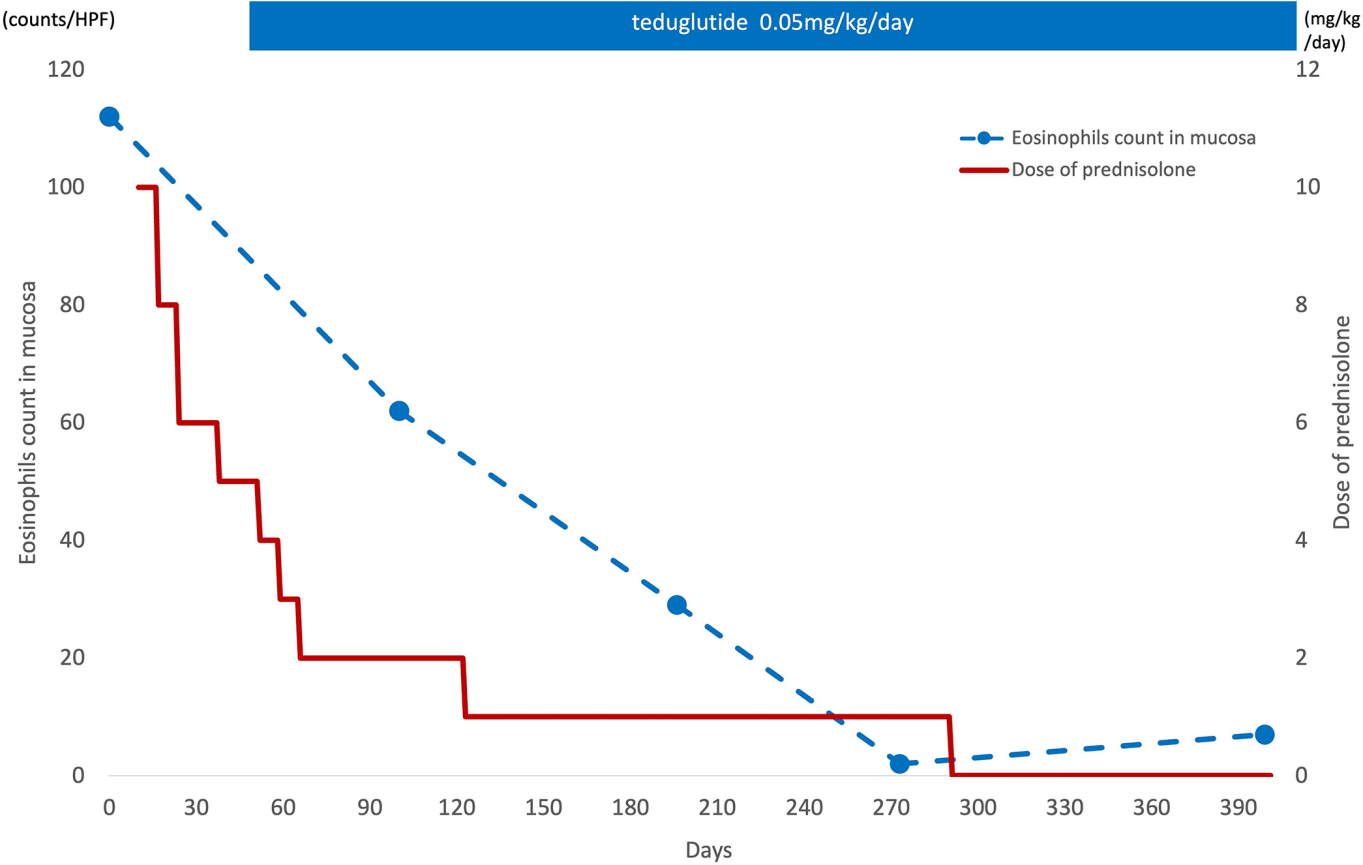
Eosinophil infiltration in the mucosa and corticosteroid therapy. After starting the teduglutide, eosinophil infiltration in the mucosa was decreased and the corticosteroid therapy could be discontinued after 6 months at the lowest dose of 1 mg/kg/day.

The patient was a girl diagnosed as having midgut volvulus at the age of 2 days and underwent resection of the strangulated intestine, which extended from 30 cm distal from the pylorus to the middle of the transverse colon. She was subsequently diagnosed as having SBS and started on parenteral nutrition. At 10 months of age, a serial transverse enteroplasty operation was performed, which increased the length of the small bowel to 62 cm. Intermittent hematochezia was observed at the age of 2 years and became persistent by the age of 4 years. Colonoscopy revealed multiple erythematous lesions in the colon and jejunum (Figure 1A). Pathological examination of these lesions showed eosinophil infiltration in the mucosa (Figure 1B), leading to a diagnosis of EGID. She had not gained sufficient weight and parenteral nutrition could not be reduced because of the SBS and EGID. Corticosteroid therapy was started at 10 mg/kg/day and gradually tapered to 1 mg/kg/day to avoid adverse effects. Although the hematochezia was slightly attenuated after the corticosteroid administration, the mucosal and pathological findings from colonoscopies did not change. At around the same time, at the age of 4 years, we started the patient on teduglutide 0.05 mg/kg once daily via hypodermic injection. Seven months later, endoscopy did not reveal any inflammation in the colon (Figure 2A), and there was no eosinophilic infiltration in the biopsy specimen (Figure 2B). We were able to discontinue the corticosteroids after 6 months at the lowest dose of 1 mg/kg/day (Figure 3). As of this writing, it has been 19 months since the patient was started on teduglutide, and her defecation pattern has changed from passing watery stool more than 10 times a day to passing soft stool 3 times a day without hematochezia. Regarding parenteral nutrition, the volume has decreased by 40% from 88 ml/kg/day to 53 ml/kg/day and the amount of calories has decreased by 37% from 46 kcal/kg/day to 29 kcal/kg/day. Her physical growth has been maintained, and her weight is now approaching the mean for her age.

## Discussion

This case suggests that administration of the GLP-2 analog teduglutide may enable discontinuation of corticosteroid therapy for EGID in SBS. In our case, histological examination confirmed almost complete disappearance of eosinophil infiltration in the intestinal mucosa after starting teduglutide as therapy for SBS.

The mechanism underlying the effect of EGID involves complex immunological cascades and networks, including immunological cells and chemical mediators such as interleukin (IL)-5 and IL-13 (8). Clinical trials are currently underway that aim to evaluate the efficacy of the biologics targeting each of the cells and mediators involved (8). Although teduglutide is reported to have pleiotropic effects on the intestine, including an anti-inflammatory action (9–11), to our knowledge, neither the specific effect on eosinophilic inflammation nor the mechanism has been reported. Meanwhile, physiological studies have reported anti-inflammatory effects of GLP-2 in the gastrointestinal tract. Specifically, GLP-2 administration has been reported to produce prominent anti-inflammatory effects in the intestinal mucosa of various mouse models of colitis (12–15) and in a postoperative ileus mouse model (16). In an animal model of obstructive jaundice with compromised intestinal barrier and immune function, Wang et al. found that serum transforming growth factor-β1 and endotoxin levels were significantly lower in rats treated with teduglutide (11). In a mouse model of total parenteral nutrition, Deng et al. demonstrated that expression levels of the inflammatory cytokines interleukin-6 and tumor necrosis factor-1α were markedly lower in mice that received teduglutide (10). In contrast, expression of antimicrobial proteins secreted by Paneth cells and proteins related to the protection and repair of intestinal epithelium was significantly increased in the teduglutide-treated group. The same study also found that the effect of teduglutide on intestinal barrier function was associated with the upregulated expression of the tight junction proteins occludin and claudin-1. In another study, it was reported that teduglutide reduces antigen uptake by enterocytes and suppresses the inflammatory response in mouse models (9). According to another report, the anti-inflammatory action of GLP-2 in the intestine is mediated by crosstalk in which enteric neurons expressing GLP-2R secrete vasoactive intestinal polypeptides, which in turn elicit anti-inflammatory effects in intestinal immune cells (15). Taken together, these reports suggest that teduglutide has effects on intestinal inflammation that contribute to the improvement of EGID.

This report has some limitations. First, we were unable to evaluate the effect of teduglutide on EGID without corticosteroid therapy, so we cannot definitively demonstrate the anti-inflammatory action of teduglutide. Second, teduglutide was used in a patient with EGID-associated SBS. Therefore, the immune-mediated effects of teduglutide when administered alone should be evaluated in patients with uncomplicated EGID. However, such a study would be difficult to perform for ethical reasons. Nevertheless, corticosteroid monotherapy did not achieve adequate remission in our patient and was successfully discontinued after administration of teduglutide. To establish evidence for the immune effects of the GLP-2 analog teduglutide, we plan to measure serum cytokines in patients treated with teduglutide and to evaluate the effect of teduglutide monotherapy on EGID in animal experiments.

In conclusion, we found that teduglutide had an anti-inflammatory effect in a patient with EGID-associated SBS. This report is the first to describe the use of teduglutide in the treatment of EGID.

## Data Availability

The original contributions presented in the study are included in the article/Supplementary Material, further inquiries can be directed to the corresponding author.
